# Effects of Modified Atmosphere Packaging with Various CO_2_ Concentrations on the Bacterial Community and Shelf-Life of Smoked Chicken Legs

**DOI:** 10.3390/foods11040559

**Published:** 2022-02-16

**Authors:** Qiang Wang, Qian Chen, Jianhang Xu, Fangda Sun, Haotian Liu, Baohua Kong

**Affiliations:** College of Food Science, Northeast Agricultural University, Harbin 150030, China; wangqiangneau@163.com (Q.W.); chenqianego7@126.com (Q.C.); xujianhangneau@163.com (J.X.); sunfangda@neau.edu.cn (F.S.); liuht920@neau.edu.cn (H.L.)

**Keywords:** smoked chicken leg, modified atmosphere packaging, CO_2_ concentration, bacterial community, single-molecule real-time sequencing

## Abstract

The effects of modified atmosphere packaging (MAP) with various CO_2_ concentrations on the bacterial community and shelf-life of smoked chicken legs during 25 d of storage at 4 °C were evaluated herein. Four treatments were stored in pallets (PAL) and MAP under 20% (M20), 60% (M60), and 100% (M100) CO_2_, respectively. The results indicated that the MAP treatments provided the legs with higher redness and hardness and lower yellowness, luminance, and lipid oxidation, compared with the PAL treatment. In addition, the MAP treatments effectively inhibited the growth of viable bacteria, delayed bacterial spoilage, and extended the shelf-life of the samples. The M60 and M100 treatments had a better inhibition effect on bacteria. In terms of bacterial community, *Carnobacterium*, *Pseudomonas*, *Brochothrix*, and *Lactococcus* were the most predominant genera in the 25 d-stored MAP samples, with *Carnobacterium maltaromaticum*, *Pseudomonas fragi*, *Shewanella baltica*, and *Lactococcus piscium* being the dominant species. However, while the inhibition effects of the M60 and M100 treatments on the bacterial community at Day 25 were similar, the outer package of the M100 treatment collapsed. Overall, the M60 treatment may be a promising approach to improving the quality and extending the shelf-life of smoked chicken legs.

## 1. Introduction

Smoked chicken leg is a very popular ready-to-eat smoked poultry product in China. As a smoked meat product, it is favored by consumers because of its attractive sugar-smoked flavor and color [1]. However, while the smoking process provides the chicken legs with a certain anti-corrosion ability, the high nutritional value and moisture content of the smoked chicken legs are easily compromised. Moreover, such products are often sold in pallets or unpackaged, thus facilitating the growth and reproduction of microorganisms, which lead to the loss of quality, e.g., slime formation, compromised texture, and an off flavor [2,3].

Modified atmosphere packaging (MAP) is a proven preservation technology for maintaining the quality and extending the shelf-life of high-grade meat and ready-to-eat meat products [4] and is widely used in the marketplace [5]. The most commonly used packaging gases for cooked meat products are carbon dioxide (CO_2_) and nitrogen (N_2_), where the CO_2_ is used to inhibit microorganisms and the N_2_ is used as the filling gas. MAP with 100% N_2_ can slow down the oxidative deterioration of food, but it has no obvious effect on inhibiting the growth of microorganisms [6], so N_2_ is generally used in combination with CO_2_. Studies have demonstrated that MAP has the ability to inhibit the growth of microorganisms when the CO_2_ concentration exceeds 20% [7]. However, while many studies have focused on the presence of spoilage microorganisms in MAP-treated meat and meat products such as sliced cooked ham [8], raw poultry sausage [9], roasted chicken [10], and precooked chicken breast slices [11], few of these have examined the effect of MAP on the structure of the microbiological community in these products. Bassey et al. [12] evaluated the effects of the gas composition on the quality characteristics and bacterial diversity in MAP fresh pork loins in chilled storage. Chen et al. [6] evaluated the effect of modified atmosphere packaging on the shelf-life and bacterial community of roasted duck legs. Moreover, second-generation sequencing technology revealed that *Pseudomonas*, *Acinetobacter*, and *Vibrio* were the main spoilage bacteria in roasted chicken [13], and *Pseudomonas*, *Weissella*, *Brochothrix*, *Lactococcus*, *Acinetobacter*, *Psychrobacter*, and *Kurthia* were the main spoilage bacteria in braised chicken [14]. To the best of our knowledge, there are no reports of applying third-generation sequencing technology to monitor the bacterial communities in meat products with MAP.

Third-generation sequencing (also known as single-molecule real-time (SMRT) sequencing) is an improved method for tapping into richer strains of bacteria. It has been shown to be more efficient and reliable than culture-dependent microbiological analysis, polymerase chain reaction-denaturing gradient gel electrophoresis (PCR-DGGE), and second-generation sequencing, for the monitoring of changes in food microbiology [15]. Compared with the other sequencing technologies, SMRT sequencing has four main advantages, namely: long read, high consistency, low deviation, and the ability to detect epigenetic characteristics. In particular, long-read SMRT sequencing enables the coverage of more repeated and missing bases and can save time by automatically eliminating gaps [16]. The SMRT sequencing technology covers both hypervariable regions V1–V9 and conserved regions, avoids primer bias, and can accurately identify the species level, which cannot be achieved by the second-generation sequencing [17].

In view of the above considerations, SMRT sequencing was used in the present study to explore the bacterial community of smoked chicken legs after MAP storage for up to 25 d at 4 °C under various concentrations of CO_2_. Meanwhile, the physicochemical properties and quality characteristics were evaluated. The identification of an optimum CO_2_ concentration is expected to provide a basis for extending the shelf-life and improving the quality of smoked meat products via MAP, and the application of SMRT sequencing will provide valuable information for the screening and targeted inhibition of spoilage bacteria.

## 2. Materials and Methods

### 2.1. Preparation of Smoked Chicken Legs

Chicken legs (thigh and drumstick) were used in this experiment. The fresh chicken legs (285 ± 10 g) and additives were purchased from Carrefour (Harbin, China). First, the fresh chicken legs were soaked in water and cleaned three times in order to remove the surface blood. The chicken legs were marinated for 12 h at 4 °C, then boiled for 50 min at 90–95 °C in a brine containing the following additives (g/L water): salt (50), ginger (2.5), garlic (1.5), onion (1.5), monosodium glutamate (1.0), liqueur (5.0), and a dry spices bag (11.5). The dry spices bag contained aniseed (2.5), pepper (2.5), orange peel (1.0), radix angelicae (1.0), cinnamon (0.5), fructus amomi (0.5), cardamom (0.5), kaempferiae galanga (1.5), and clove (1.5). After cooling to room temperature, the cooked chicken legs were smoked in the sugar smoking device described previously [1]. For this process, cooked chicken legs were placed on a stainless-steel tray with small holes. When the temperature of the iron pot bottom reached 200–210 °C, 50.0 g of sucrose was placed in the bottom of the iron pot. Then, the tray with chicken legs was placed on the pot, the lid was immediately positioned, and the smoking was performed for 4 min. The smoked chicken legs were then cooled to room temperature (20 ± 5 °C) and subjected to MAP once the color was stable.

### 2.2. Packaging and Storage

Four distinct packaging treatments were applied, including palletization (PAL) and MAP under 20% (M20), 60% (M60), and 100% (M100) CO_2_. In the PAL treatment, each smoked chicken leg was individually placed in a polypropylene tray (TQBC-0775, Sealed Air Corp., Danbury, CT, USA) and then covered with an oxygen-permeable polyethylene film (8 μm in thickness and 18,500 cm^3^ m^−2^ per 24 h of oxygen permeability; Weiguang Plastic Co., Ltd., Shanghai, China). In the MAP treatments, for the M20 and M60 samples, the CO_2_ was mixed with N_2_. Each smoked chicken leg was individually placed in a polypropylene tray (TQBC-0775, Sealed Air Corp., Danbury, CT, USA), which was then flushed with the desired gas mixture and sealed with an oxygen-barrier film (Lid 1050, Sealed Air Corp., Danbury, CT, USA) using a DT-6D packaging machine (Dajiang Machinery Equipment Co., Ltd., Wenzhou, China). According to the supplier specifications, the polypropylene trays have an oxygen transmission rate of 10 cm^3^ m^−2^ per 24 h at 23 °C and 0% relative humidity (RH) and a water vapor transmission rate of 15 g m^−2^ per 24 h at 38 °C and 90% RH, while the oxygen-barrier film has an oxygen transmission rate of 25 cm^3^ m^−2^ per 24 h at 23 °C and 0% RH and a water vapor transmission rate of 10 g m^−2^ per 24 h at 4 °C and 100% RH. After packaging, all legs (100 packaged and 5 unpackaged) were stored at 4 °C. After 2 h, the 5 unpackaged legs were used as the Day 0 samples. There were 105 chicken legs used for each batch production. Five of them were used at 0 d and the other one-hundred packaged samples were used for the storage experiment. Each treatment had 25 samples. Samples were stored for 0 d, 5 d, 10 d, 15 d, 20 d, and 25 d. For each treatment at each storage time (5 d, 10 d, 15 d, 20 d, and 25 d), five smoked chicken legs were used for the analysis. Therefore, in total, there were 315 chicken legs used for 3 production batches. The thighs were used to analyze the physicochemical properties, and the drumsticks were used to evaluate the total viable counts (TVC) and for DNA extraction and sequencing. For the physicochemical properties, first, the hardness was measured, then the surface skin color was analyzed, and finally, the pH value, moisture content, and lipid oxidation were determined. However, the PAL-packaged legs were spoiled after 15 d of storage and were not examined thereafter. In addition, for bacterial community analysis, the legs were sampled at 0 d and the MAP legs at 25 d.

### 2.3. Determination of the pH Value and Moisture Content

The smoked chicken sample (10.0 g) was mixed with 90.0 mL of 0.1 mol/L KCl and homogenized at 10,000 rpm for 60 s. The pH of the mixture was then determined using a pH meter (Mettler Toledo Instruments Co., Ltd., Shanghai, China) in accordance with the procedure of Lv et al. [18]. In addition, the moisture content was measured according to the Association of Official Analytical Chemists (AOAC) standard procedure [19]. Two grams of minced chicken was evenly spread on the bottom of the aluminum dish, then dried in an oven (101-3S air oven Lichen Science and Technology Co., Ltd., Shanghai, China) at 102 °C for 16 h. The moisture content was calculated as the weight of the lost moisture as a percentage of the weight of the sample.

### 2.4. Determination of Lipid Oxidation

Based on the method of Chen et al. [20], the content of lipid oxidation was evaluated according to the thiobarbituric acid reactive substance (TBARS) value:

TBARS (mg/kg) = (*A*_532_/*W*) × 9.48 (1)

where *A*_532_ is the absorbance of the assay solution at 532 nm, *W* is the weight of the sample (g), and 9.48 is a constant derived from the dilution factor and the molar extinction coefficient (152,000 M^−1^ cm^−1^) of the thiobarbituric acid reaction product.

### 2.5. Determination of Hardness

A TA-XT plus texture analyzer (Stable Micro Systems, Godalming, UK) with a Meullenet–Owens razor shear (MORS) probe was used to determine the texture profile of the smoked chicken legs. The hardness was determined using the textural profile analysis (TPA) mode, with a pre-test speed of 2 mm/s, a triggering force of 20 g, and a compression distance of 10 mm [21]. The thighs with bones were placed on the center of the console and cut six times for the hardness measurement. The values are expressed as newton (N) units.

### 2.6. Determination of Color

The color on the skin of the chicken leg was measured using a ZE-6000 colorimeter (Juki Corp., Tokyo, Japan) with a D65 light source and a 10° observer. The values were expressed as the L*-value (lightness), a*-value (redness), and b*-value (yellowness). A white standard plate (L* = 95.26, a* = −0.89, b* = 1.18) was used for calibration.

### 2.7. Determination of Total Viable Counts

According to GB 2726-2016 [22], the packages containing the smoked chicken legs were aseptically opened, the chicken legs (minus the bones) were chopped, then 25.0 g samples were homogenized in sterilized 0.85% NaCl (225 mL) solution, followed by a series of dilutions with sterilized 0.85% NaCl (9.0 mL) solution. Each dilution was then inoculated into plate count agar (PCA, Hope Bio-Tech, Qingdao, China) and incubated at 37 ± 1 °C for 48 h to determine the TVC. The results are expressed as log CFU/g chicken legs.

### 2.8. DNA Extraction and Sequencing

#### 2.8.1. Extraction of Bacterial DNA

The bacterial DNA was extracted from the smoked chicken legs (0.2 g) using the cetyl trimethyl ammonium bromide/sodium dodecyl sulphate (CTAB/SDS) method [23]. The DNA purity was determined using 1% (*w*/*v*) agarose gel, and the DNA concentration was diluted to 1 ng/μL using sterile water. The DNA concentration and purity were determined using Nanodrop 2000 (Thermo Fisher Scientific, Waltham, MA, USA) to ensure the OD_260/280_ was between 1.8 and 2.0. The cetyl trimethyl ammonium bromide/sodium dodecyl sulphate and agarose gel were obtained from Solarbio Technology Co. Ltd. (Beijing, China).

#### 2.8.2. Amplification and Sequencing of 16S rRNA

The bacterial 16S rRNA was amplified via the polymerase chain reaction (PCR) according to previously reported conditions and methods [23]. The V1–V9 region of the bacterial 16S rRNA gene was amplified using the specific primers with barcodes (forward: 5′-AGAGTTTGATCCTGGCTCAG-3′; reverse: 5′-GNTACCTTGTTACGACTT-3′). The PCR was performed using TransStart^®^ FastPfu DNA Polymerase (TransGen Biotech, Beijing, China) according to the following protocol: (i) initial denaturation at 98 °C for 5 min, (ii) 35 cycles of denaturation at 95 °C for 30 s, (iii) annealing at 60 °C for 45 s, (iv) extension at 72 °C for 9 s, and (v) extension at 72 °C for 10 min. The PCR products were detected via 2% agarose gel electrophoresis and purified using a QIAquick@ gel extraction kit (QIAGEN, Hilden, Germany). The sequencing libraries were generated using the SMRTbell TM Template Prep Kit (Pacific Biosciences, Menlo Park, CA, USA). The library quality was assessed on the Qubit@ 2.0 Fluorometer (Thermo Fisher Scientific, Waltham, MA, USA) and the FEMTO Pulse system. Lastly, the library preparation and sequencing were conducted with the help of Novogene Company (Beijing, China, https://www.novogene.com/ (6 May 2021)).

#### 2.8.3. Bioinformatics Analysis

The raw reads were filtered and analyzed using the QIIME software package (Version 1.9.1). During this process, the UCHIME algorithm (http://www.drive5.com/usearch/manual/uchime_algo.html (20 May 2021)) was used to eliminate any ambiguous and chimeric sequences, and the clean reads were validated by comparison with the Gold database (http://drive5.com/uchime/uchime_download.html (23 May 2021)). The sequence analysis was performed using the Uparse software (Uparse v7.0.1001, http://drive5.com/uparse/ (23 May 2021)). Sequences with ≥97% similarity were assigned to the same operational taxonomic unit (OTU), and representative sequences were then screened for each OTU. For each representative sequence, the Silva SSUrRNA database (https://www.arb-silva.de/ (28 May 2021)) was used based on the Mothur algorithm for annotating the taxonomic information. The alpha-diversity (Shannon, Simpson, abundance-based coverage estimator (ACE), Chao1, Good’s coverage) and beta diversity indices on the weighted and unweighted UniFrac distances were calculated using the QIIME software (Version 1.7.0). To assess the differences between the bacterial communities of the MAP smoked chicken legs with various concentrations of CO_2_, a principal co-ordinates analysis (PCoA) was performed on the identified OTU, and the results were displayed using the R software (Version 2.15.3). Then, the 30 most abundant bacterial species were selected as the core bacteria for correlation by distance-based redundancy analysis (db-RDA).

### 2.9. Statistical Analysis

Three independent batches (replicates) of the smoked chicken legs were prepared, and all measurements were conducted in triplicate for each batch. The data were analyzed via the General Linear Models procedure of the Statistix 8.1 software package (Analytical Software, St. Paul, MN, USA), and the results are expressed as the mean ± standard error (SE). The normality and homogeneity of variance were analyzed via the Shapiro–Wilk and Levene procedure of the Statistix 8.1 software package (Analytical Software, St Paul, MN, USA), *p* > 0.05. The significance of each treatment effect (*p* < 0.05) was evaluated via an analysis of variance (ANOVA) and Tukey’s multiple comparison test, and the data were plotted using the Origin 2019 software package (Analytical Software, Systat, Hampton, MA, USA). In addition, the db-RDA correlation was performed and plotted using the R software (Version 2.15.3) package.

## 3. Results and Discussion

### 3.1. pH Value and Moisture Content

**Figure 1:**
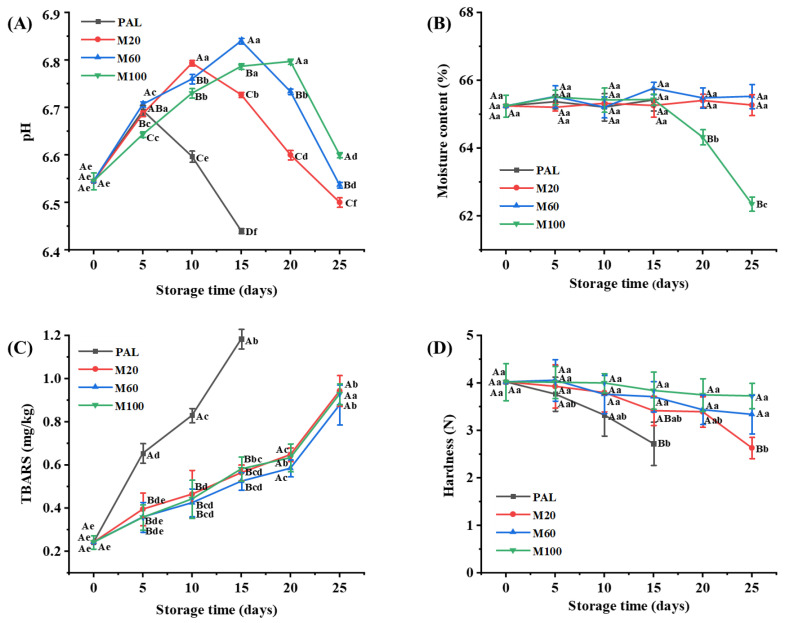
The changes in pH (**A**), moisture content (**B**), thiobarbituric acid reactive substances (TBARS) (**C**), and hardness (**D**) of the variously packaged smoked chicken legs during storage for up to 25 d at 4 °C. PAL: the palletized smoked chicken legs; M20, M60, and M100: the modified atmosphere packaged smoked chicken legs with CO_2_ concentrations of 20%, 60%, and 100%, respectively. The different uppercase letters (A–D) indicate significant differences between the variously packaged samples after the same storage time (*p* < 0.05), while the different lowercase letters (a–f) indicate significant differences in the same packaged sample after different storage times (*p* < 0.05).

The changes in the pH and moisture content of the variously packaged smoked chicken legs during storage for up to 25 d at 4 °C are presented in Figure 1A,B, respectively. For all treatments, the pH was seen to initially increase, then decrease, within the range of 6.44 to 6.84 (Figure 1A). It is generally believed that the antibacterial effect of CO_2_ is mainly dependent on its dissolution in water to form H_2_CO_3_, which dissociates into H^+^ and HCO_3_^−^ ions, thus reducing the pH. The observed, and contrary-to-expected, increase in the initial pH of the various samples may be attributed to the following two factors: (i) the anaerobic environment with a high concentration of CO_2_ promoted the expression of arginine deiminase, alanine dehydrogenase, and tyrosine dehydrogenase in microorganisms such as *Carnobacterium*, which catalyze the production of ammonia and biogenic amines, thus maintaining the pH stability and weakening the antibacterial effect of the CO_2_ [24]; (ii) the accumulation of ammonia from the decomposition of nucleic bases and amino acid during the whole stages of the storage can lead to an increase in pH [11]. During the later stages of storage, the decrease in the pH values may be related to a significantly increased relative abundance of acid-producing microorganisms. Notably, the highest pH values were observed on Days 5, 10, 15, and 20 for the PAL, M20, M60, and M100 samples, respectively (*p* < 0.05). Thus, the increase in CO_2_ concentration during packaging was seen to delay the decrease in pH. This, in turn, can potentially decrease the rate of spoilage by inhibiting the expression of metabolic proteins in lactic acid bacteria, thus reducing the production of lactic acid [11].

No significant change in the moisture content was observed for the PAL, M20, and M60 samples during storage (*p* > 0.05), thus indicating that MAP with medium and low concentrations of CO_2_ does not affect the moisture content (Figure 1B). By contrast, the moisture content of the M100 sample was seen to significantly decrease from 65.43% on Day 15 to 62.35% on Day 25 (*p* < 0.05), at which time the outer package was seen to be seriously collapsed and deformed. This may be because some of the CO_2_ content became dissolved in the fat and water [12], thus generating negative pressure and causing the package to dent noticeably. In addition, the spoilage process is accompanied by the reproduction of microorganisms and the oxidation of fat and protein [6], which may lead to the quality deterioration of smoked chicken legs. Under negative pressure, the moisture content of the smoked chicken legs could flow out and be seen in the package. Such a collapse and deformation of the outer package can seriously affect product sales. Indeed, similar results were previously reported by Sun et al. [25], who observed high drip loss, serious collapse, and a low overall acceptability of swimming crab packaged in 100% CO_2_.

### 3.2. Lipid Oxidation

The changes in the content of TBARS of the variously packaged smoked chicken legs during storage for up to 25 d at 4 °C are presented in Figure 1C. Here, the TBARS value of the PAL sample was seen to increase significantly from 0.24 on Day 0 to 1.18 on Day 15, while that of the M20, M60, and M100 samples reached 0.94, 0.88, and 0.93, respectively, on Day 25 (*p* < 0.05). Thus, the TBARS value of the PAL sample was significantly higher than that of the MAP samples during storage (*p* < 0.05). This may be because lipid oxidation was promoted by the presence of O_2_ in the PAL treatment, but inhibited by the presence of CO_2_ in the MAP treatments. Similar results were obtained by Guo et al. [7], who found that an anaerobic environment can inhibit the lipid oxidation of roasted chicken. Moreover, Del Olmo et al. [26] found that the combination of CO_2_ and N_2_ can minimize the oxidative deterioration of cured/cooked pork meat products.

### 3.3. Hardness

Hardness is a very important quality indicator of cooked meat products. As shown in Figure 1D, the hardness of the fresh smoked chicken legs on Day 0 was the highest (at 4.02 N) and decreased to 2.85 N for the PAL sample on Day 15 and to 2.63, 3.34, and 3.73 N for the M20, M60, and M100 samples, respectively, on Day 25 (*p* < 0.05). This may be attributed to the deterioration of the product structure due to the reproduction of microorganisms and oxidation of the product [27]. It can also be noted that, on day 15, the hardness of the M60 and M100 samples was higher than that of the M20 sample, and the hardness of the M20 sample was significantly higher than that of the PAL sample (*p* < 0.05). Furthermore, on Day 25, the hardness of the M60 and M100 samples was significantly higher than that of the M20 sample (*p* < 0.05). These results indicate that the MAP treatments with higher concentrations of CO_2_ provided better protection to the texture of the chicken legs. Notably, the hardness of the M100 sample did not increase during storage, even though its moisture content decreased significantly from 65.43% to 62.35%.

### 3.4. Color

The color of the skin is a key factor in the customers’ decision to purchase the product and is an important indicator of freshness. The changes in the skin color of the smoked chicken legs during storage for up to 25 d at 4 °C are indicated in Table 1. Here, the yellowness (b*-value) of each sample was seen to increase from 39.22 at Day 0 to 41.74 for the PAL sample on Day 15 and to 42.34–42.55 for the various MAP samples on Day 25 (*p* < 0.05). This may be due to the generation of a yellow pigment by the reaction of the lipid oxidation products with amines in the phospholipid head groups or in the proteins [18].

Meanwhile, the redness (a*-value) was seen to decrease from 18.06 on Day 0 to 14.9 on Day 15 for the PAL sample and to 15.39–15.78 on Day 25 for the MAP samples (*p* < 0.05). The decrease in redness may be due to the interaction between the pigment and the lipid oxidation products [28]. Similar results were reported by Chen et al. [6], who demonstrated that lipid oxidation can lead to the decrease of the a*-value of the skin surface of roasted duck legs with MAP. Moreover, the MAP samples each showed a relatively stable redness after Day 5. Similarly, Chen et al. [6] demonstrated that the a*-value of roasted duck legs with MAP (100% N_2_, 30% CO_2_/70% N_2_, 50% CO_2_/50% N_2_, and 0.4% CO/30% CO_2_/69.6% N_2_) was significantly higher than that of the air package.

Finally, the lightness (L*-value) of the PAL sample was seen to increase from 38.54 on Day 0 to 40.62 on Day 5 and to 42.06 on Day 15. The initial increase may be due to the presence of moisture on the skin of the legs caused by the low temperature and high humidity [10]. Meanwhile, the MAP samples each exhibited a relatively stable L*-value, with a slight increase from 38.54 on Day 0 to 39.70–40.60 on Day 25. Notably, on Day 15, the L*-value of the PAL sample (i.e., 42.06) was higher than that of the MAP samples (39.75–40.36; *p* < 0.05), which may be due to the more intense oxidative cleavage of pigment in the PAL sample [29], which is consistent with the changing trend of the a*-value. These results indicate that MAP is an efficient method for maintaining the skin color of smoked chicken and are consistent with the results of Chen et al. [6] and Guo et al. [7] for the benefits of MAP for roasted chicken and duck.

### 3.5. Total Viable Counts

**Figure 2:**
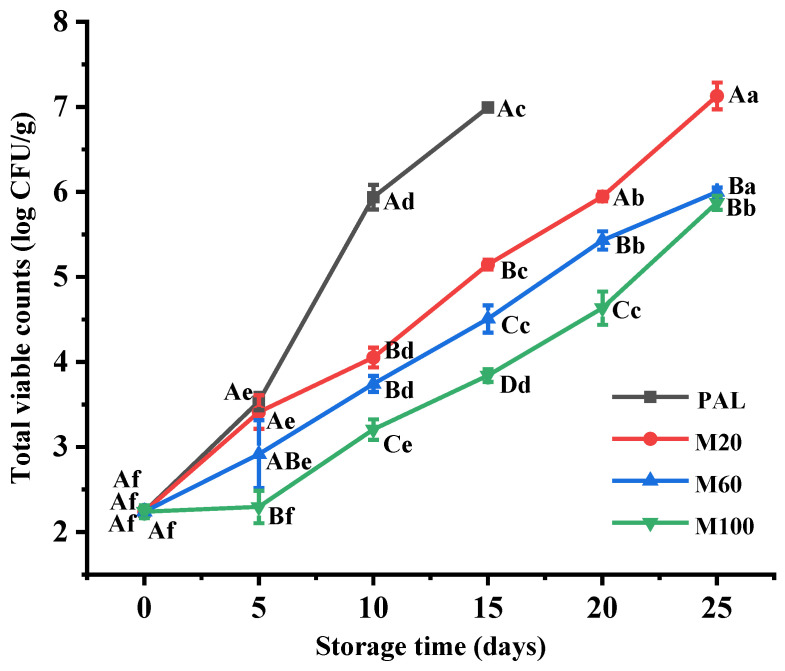
The changes in the total viable counts of the variously packaged smoked chicken legs during storage for up to 25 d at 4 °C. PAL: the palletized smoked chicken legs; M20, M60, and M100: the modified atmosphere packaged smoked chicken legs with CO_2_ concentration of 20%, 60%, and 100%, respectively. The different uppercase letters (A–D) indicate significant differences between the variously packaged samples after the same storage time (*p* < 0.05), while the different lowercase letters (a–f) indicate significant differences in the same packaged sample after different storage times (*p* < 0.05).

The changes in the TVC of the variously packaged smoked chicken leg samples during storage for up to 25 d at 4 °C are presented in Figure 2. For the PAL sample, the TVC was seen to increase significantly from 2.24 log CFU/g on Day 0 to 6.99 log CFU/g on Day 15, at which time, the storage was terminated due to evident spoilage. Even on Day 10, the TVC of the PAL sample (i.e., 5.94 log CFU/g) was significantly higher than that of the MAP samples (3.21–4.05 log CFU/g; *p* < 0.05). Thereafter, the TVC values of the M20, M60, and M100 samples continued to increase up to 7.13 log CFU/g, 6.00 log CFU/g, and 5.87 log CFU/g, respectively, on Day 25 (*p* < 0.05). These results indicate that MAP provides a better inhibitory effect on bacterial growth and that this effect increases with the increase in CO_2_ concentration. According to GB 2726-2016 [22], the safety limit value of TVC is ≤5 log CFU/g for cooked meat products. During storage, the TVC values of the PAL, M20, M60, and M100 samples were more than 5 log CFU/g on Days 10, 15, 20, and 25, respectively. Except for the PAL sample on Day 15, the TBARS values of all the samples were lower than 1.0 mg MDA/kg (a threshold value for perceiving undesirable rancid flavor and odor) during storage (Figure 1C), indicating that such a degree of lipid oxidation has no negative effect on the sensory quality of the products [21]. The hardness of the PAL samples decreased obviously on Day 10, which may be due to the breakdown of the muscle tissue structure caused by microbial growth. In addition, the changes in the moisture content, pH, and color of all samples during storage were within an acceptable range. Although the M100 samples had the lowest TVC and least quality deterioration during storage, the serious collapse of the outer packaging induced by the high CO_2_ concentration may cause a decrease in the acceptability of the product. In general, the shelf-life of products is affected by many factors, and the shelf-life of the MAP samples was significantly longer than that of the PAL samples. The shelf-life of the MAP treatments in this study was shorter than that reported by Guo et al. [7], which may be due to the higher initial TVC level and the different cooking methods.

### 3.6. Bacterial Community Diversity Analysis

#### 3.6.1. Bacterial Diversity and Richness

The bacterial diversities of the variously packaged smoked chicken legs after 25 d of storage at 4 °C are indicated in Table 2. For comparison, the bacterial diversity of a control sample (the fresh, unpacked chicken legs on Day 0) is also indicated. Here, the number of high-quality clean reads obtained for each sample ranged from 7207 to 9584, with an average of 8682.5. The richness (ACE and Chao1) indices and observed operational taxonomic units (OTUs) of the control sample were higher than those of the variously packaged samples at Day 25. Furthermore, the diversity (Shannon and Simpson) indices of the M20 sample were lower than those of the M60 and M100 samples. Finally, the Good’s coverage was above 99.3% for all samples, thus indicating that most of the bacterial phylotypes were detected.

#### 3.6.2. Bacterial Community

**Figure 3:**
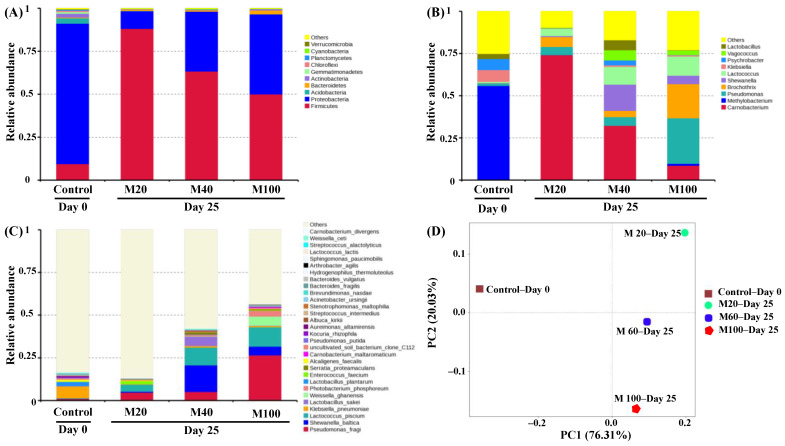
The relative bacterial abundances at (**A**) the phylum level, (**B**) the genus level, and (**C**) the species level for the control sample (fresh smoked chicken legs, Day 0) and the variously packaged samples at Day 25 and (**D**) the corresponding principal co-ordinates analysis. M20, M60, and M100: the modified atmosphere packaged smoked chicken legs with CO_2_ concentration of 20%, 60%, and 100%, respectively.

The SMRT sequencing analyzed the bacterial community in the smoked chicken legs well and realized the species-level identification. Moreover, such an extended read length enabled higher confidence in the phylogenetic classifications and offered additional OTU for clusterin improvement, effectively reducing the underestimation or overestimation of the taxonomic diversity occasionally caused by second-generation sequencing [17]. The relative bacterial abundances in the smoked chicken leg samples at Day 0 and Day 25 are presented at the phylum, genus, and species levels, along with a principal co-ordinates analysis, in Figure 3. Here, a total of 12 phyla were identified in the sequencing analysis (Figure 3A), with *Firmicutes* and *Proteobacteria* predominating at the beginning and end of storage. This is comparable to the results obtained by Zhang et al. [30] for cooked meat. In the present study, the relative abundance of *Proteobacteria* (81.71%) was higher than that of *Firmicutes* (9.46%) in the fresh smoked chicken legs (control, Day 0), followed by *Acidobacteria* (3.14%) and *Actinobacteria* (2.21%). By contrast, the relative abundance of *Firmicutes* dramatically increased in the MAP samples on Day 25, when the relative abundance of *Firmicutes* was 88.14%, 63.33%, and 50.01% in the M20, M60, and M100 samples, respectively. Conversely, the relative abundance of *Proteobacteria* was seen to decrease to 10.23%, 34.81%, and 46.54% in the M20, M60, and M100 samples, respectively. Thus, the relative abundance of *Firmicutes* was negatively correlated with the concentration of CO_2_ in the packaging treatment, whereas the relative abundance of *Proteobacteria* was positively correlated with the concentration of CO_2_. In other words, these results demonstrate that an increased CO_2_ concentration can inhibit *Firmicutes* and promote *Proteobacteria*. Similar trends in *Firmicutes* and *Proteobacteria* were previously observed by Chen et al. [6] in fresh roasted duck meat and in MAP roasted duck meat with various CO_2_ concentrations, after storage for 7 d and 14 d.

At the genus level, 103 genera were identified, with *Carnobacterium*, *Methylobacterium*, *Pseudomonas*, *Brochothrix*, *Shewanella*, and *Lactococcus* being the most prevalent in all samples (Figure 3B). In the fresh smoked chicken legs, *Methylobacterium* was the predominant genus (55.59%), followed by *Klebsiella* (6.98%), and *Psychrobacter* (6.57%). *Methylobacterium* is ubiquitous in nature and exists in various environments (such as soil, dust, fresh water, and laboratory environment) [31]. *Klebsiella* is a conditional pathogen that can cause pneumonia and widely exists in chickens, sheep, pigs, and other animals [32]. *Psychrobacter* is an aerobic bacterium that can grow readily under chilled conditions and has been identified as the main genus in roasted chicken that has been packaged in air [13].

For the three MAP treatments, significant differences were observed in the bacterial community at the genus level on Day 25, thus revealing the influence of the atmospheric composition used in the MAP. Overall, *Carnobacterium*, *Pseudomonas*, *Brochothrix*, *Shewanella*, and *Lactococcus* were the predominant genera. This is consistent with other recent studies, where the predominance of *Lactobacillus*, *Brochothrix*, *Pseudomonas*, and *Carnobacterium* has been associated with the spoilage of MAP meats [16,33]. In detail, the main genera in the M20 sample on Day 25 were *Carnobacterium* (74.15%), *Brochothrix* (5.85%), *Pseudomonas* (4.86%), and *Lactococcus* (4.28%), while the main genera in the M60 sample were *Carnobacterium* (32.28%) and *Shewanella* (15.59%), followed by *Lactococcus* (10.48%), *Vagococcus* (6.01%), *Lactobacillus* (5.91%), *Pseudomonas* (5.16%), and *Brochothrix* (3.70%). Further, relatively high abundances of 27.01%, 20.19%, 11.37%, and 8.63% were observed for *Pseudomonas*, *Brochothrix*, *Lactococcus*, and *Carnobacterium*, respectively, in the M100 sample on Day 25.

The results in Figure 3B reveal that the relative abundance of *Carnobacterium* was negatively correlated with the concentration of CO_2_ in the packaging treatment, thus indicating that CO_2_ has a significant inhibitory effect on *Carnobacterium*. This is consistent with the results of Zhang et al. [30], who reported that *Carnobacterium* was the dominant genus on chilled beef primal cuts collected from three Canadian abattoirs at the end of storage. However, while the growth of *Carnobacterium* has a minor impact on the sensory properties of meat and meat products, the presence of *Brochothrix* can produce an off odor and slime [34].

In the present study, the abundance of *Pseudomonas* and *Lactococcus* was positively correlated with the CO_2_ concentration in the packaging treatment. This result was unexpected for *Pseudomonas* because it is sensitive to CO_2_; nevertheless, Chen et al. [6] also identified high levels of *Pseudomonas* in roasted chicken that were stored in MAP under a high concentration of CO_2_ (50%), thus indicating incomplete inhibition of this genus. Previous studies have also identified *Lactococcus* as a dominant bacterium and a cause of reduced shelf-life in various MAP meat products, including chicken, pork, and beef [35].

In Figure 3B, the 25 d-stored M100 samples exhibited the highest abundance of *Brochothrix* out of all the MAP samples. As a facultatively anaerobic bacterium, *Brochothrix* is commonly found at high levels in meat and meat products under various packaging conditions and is the dominant spoilage species in roasted duck meat [6]. Meanwhile, the 25 d-stored M60 sample exhibited the highest abundance of *Shewanella*. This is a group of Gram-negative, facultative anaerobic, H_2_S-producing rod-shaped bacteria that are responsible for spoilage by producing a “fishy” off odor [36].

At the species level, a total of 62 species were identified in all the samples, with *Pseudomonas fragi*, *Shewanella baltica*, *Lactococcus piscium*, *Klebsiella pneumoniae*, and *Lactobacillus sakei* predominating (Figure 3C). In the fresh smoked chicken legs, *K. pneumoniae* is the predominant species (6.98%), followed by *Lactobacillus plantarum* (2.48%), and *Alcaligenes faecalis* (1.30%). In the 25 d-stored M20 sample, however, the main species were *P. fragi* (4.74%), *L. piscium* (4.11%), and *Enterococcus faecium* (1.68%). For the M60 sample, *S. baltica* (15.59%) and *L. piscium* (10.32%) were the main species at the end of storage, followed by *L. sakei* (5.43%), *P. fragi* (5.16%), and *Serratia proteamaculans* (1.38%). Finally, for the M100 sample, the predominant species were *P. fragi* (26.62%), *L. piscium* (11.31%), *Weissella ghanensis* (5.41%), *S. baltica* (4.99%), and *Photobacterium phosphoreum* (4.99%). These results reveal an increase in the relative abundances of *P. fragi* and *L. piscium* with increased CO_2_ concentration in the MAP treatments. Among the psychrotrophic spoilage *Pseudomonas* species, *P. fragi* is the most frequently encountered on meats such as beef, chicken, pork, and lamb [37]. Although CO_2_ increases the permeability of the *P. fragi* cell membrane and may alter the ability of the cell to absorb ions by inhibiting the expression of certain transporters, it does not destroy the cell integrity [38]. *P. fragi* readily forms a surface biofilm that can combine with the meat exudate to generate slime, which is a key quality defect that can influence consumer acceptability [39]. In addition, *P. fragi* can promote the release of amines and increase the pH [40]. Meanwhile, the growth of *L. piscium* can lead to meat spoilage and decreased shelf-life [35]. In addition, *S. baltica* is a major spoilage bacterium in seafood and produces volatile compounds with a spoilage smell [41]. By contrast, *L. sakei* and *L. plantarum* can produce lactic acid, hydrogen peroxide, bacteriocin, and other bacteriostatic metabolites in the later stages of low-oxygen MAP storage, thus inhibiting the growth of other bacteria and prolonging the shelf-life [42]. Notably, just as *Carnobacterium* exhibited the highest abundance at the genus level, *Carnobacterium divergens* and *Carnobacterium maltaromaticum* were identified at the species level. These two species can maintain their homeostasis and structural stability by regulating their protein expression and metabolic activities so as to adapt to conditions of 20–40% CO_2_ [24,43]. Furthermore, they can promote the expression of arginine deiminase, alanine dehydrogenase, and tyrosine dehydrogenase and can catalyze the production of alkaline products such as ammonia and biogenic amines to maintain the stability of their internal environment. Moreover, *C. maltaromaticum* also promotes the expression of acetyl-CoA carboxylase, S-malonyl transferase, and 3-keto acyl ACP reductase related to fatty acid synthesis of the cell membrane and regulates the fluidity of the cell membrane in order to maintain its structural stability in conditions of 20–40% CO_2_ [24].

#### 3.6.3. Differences in the Bacterial Composition

The results of the PCoA analysis are presented in Figure 3D; two principal components (labelled PC1 and PC2) accounted for 96.34% of the total variance. Further, the 25 d-stored M20, M60, and M100 samples exhibited relatively similar PC1 values that differed significantly from that of the fresh smoked chicken leg sample on Day 0. Moreover, the PC1 value of the M60 sample was much more similar to that of the M100 sample than to the M20 sample, thus indicating similar bacterial communities in the M60 and M100 samples in the later stages of storage. It seems that the MAP with various concentrations of CO_2_ would significantly influence the bacterial communities [6].

### 3.7. The Correlations between Bacterial Community and Environmental Indicators

**Figure 4:**
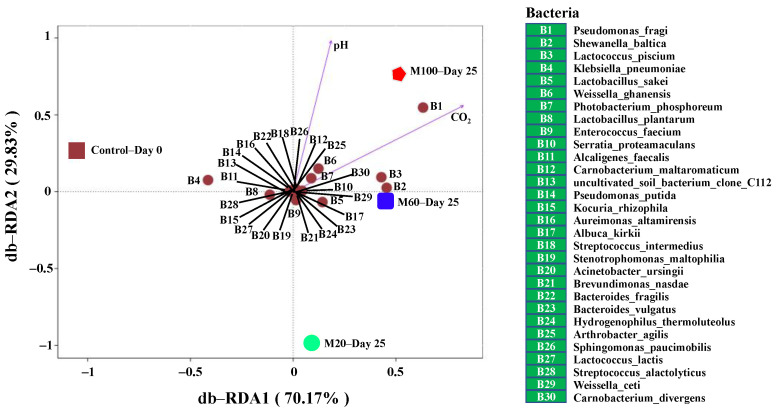
A distance-based redundancy analysis (db-RDA) of the correlations between the 30 most dominant bacteria and the environmental factors (pH and CO_2_ concentration) for the control sample (fresh smoked chicken legs, Day 0) and the variously packaged samples at Day 25. M20, M60, and M100: the modified atmosphere packaged smoked chicken legs with CO_2_ concentration of 20%, 60%, and 100%, respectively.

The CO_2_ concentration and pH are important environmental indicators that affect bacterial communities. Hence, the db-RDA correlations between the 30 most dominant bacteria and the environmental factors (CO_2_ and pH) of the variously packaged smoked chicken legs are presented in Figure 4. Here, the relative abundance of each bacterium (B1–B30) is indicated by its distance from the origin, and the correlation between the bacterial abundance and each environmental factor is indicated by the relative orientation of the point representing the bacterium with respect to the arrowed line representing the specific environmental indicator. Thus, *P. fragi*, *S. baltica*, *L. piscium*, *W. ghanensis*, and *P. phosphoreum* (B1, B2, B3, B6, and B7, respectively) were significantly positively correlated with CO_2_ and pH (appearing in the same quadrant of the plot), thereby indicating that these strains can survive in high concentrations of CO_2_ [6,13]. Furthermore, the point B1 was closest to the red pentagon representing the M100 treatment, thereby indicating that *P. fragi* was the most abundant species in the M100 sample on Day 25. In addition, the closeness of points B2 and B3 to the blue quadrangle representing the M60 treatment indicates that *S. baltica* (B2) and *L. piscium* (B3) were abundant in the M60 treatment on Day 25. These results are consistent with the above-mentioned relative abundance analysis and with the OTU-based PCoA, thus confirming that similar bacterial communities developed in the chicken legs stored under 60% CO_2_ (M60) and 100% CO_2_ (M100).

## 4. Conclusions

The present study examined the effects of MAP under various CO_2_ concentrations on the bacterial community and shelf-life of smoked chicken legs. The MAP treatments provided better color, hardness, and pH stability during storage, along with lower lipid oxidation and total viable counts, than did the PAL treatment. In addition, the MAP treatment effectively delayed the onset of bacterial spoilage and extended the shelf-life of the smoked chicken legs. The MAP treatments with 60% and 100% CO_2_ had similar inhibitory effects on the bacterial community and similar effects on the total viable counts after storage at 4 °C for 25 d. However, the MAP treatment with 100% CO_2_ resulted in severe denting of the package and a decreased moisture content of the smoked chicken legs at the later stages of storage. In conclusion, the MAP treatment with 60% CO_2_ is recommended as a promising packaging method for improving the quality of smoked chicken legs.

## Figures and Tables

**Table 1:** The changes in color of the variously packaged smoked chicken legs during storage at 4 °C.

Storage Time	Treatment			
	PAL	M20	M60	M100
L*-value				
Day 0	38.54 ± 0.96 ^Ab^	38.54 ± 0.96 ^Aa^	38.54 ± 0.96 ^Aa^	38.54 ± 0.96 ^Aa^
Day 5	40.62 ± 0.93 ^Aab^	40.79 ± 1.08 ^Aa^	40.45 ± 0.98 ^Aa^	40.16 ± 1.14 ^Aa^
Day 10	40.74 ± 0.66 ^Aab^	40.59 ± 0.88 ^Aa^	39.66 ± 0.91 ^Aa^	40.36 ± 0.43 ^Aa^
Day 15	42.06 ± 0.90 ^Aa^	40.27 ± 0.64 ^Ba^	40.36 ± 0.75 ^Ba^	39.75 ± 0.27 ^Ba^
Day 20		39.82 ± 0.75 ^Aa^	40.59 ± 0.63 ^Aa^	40.50 ± 0.63 ^Aa^
Day 25		40.60 ± 0.61 ^Aa^	39.70 ± 0.40 ^Aa^	40.41 ± 0.91 ^Aa^
a*-value				
Day 0	18.06 ± 0.56 ^Aa^	18.06 ± 0.56 ^Aa^	18.06 ± 0.56 ^Aa^	18.06 ± 0.56 ^Aa^
Day 5	16.39 ± 0.44 ^Ab^	16.27 ± 0.55 ^Ab^	16.53 ± 0.51 ^Ab^	16.20 ± 0.87 ^Ab^
Day 10	15.63 ± 0.51 ^Abc^	16.19 ± 0.55 ^Ab^	16.23 ± 0.52 ^Ab^	16.00 ± 0.74 ^Ab^
Day 15	14.90 ± 0.62 ^Ac^	15.88 ± 0.45 ^Ab^	16.05 ± 0.63 ^Ab^	15.75 ± 0.35 ^Ab^
Day 20		15.71 ± 0.72 ^Ab^	15.84 ± 0.41 ^Ab^	15.55 ± 0.40 ^Ab^
Day 25		15.39 ± 0.50 ^Ab^	15.78 ± 0.43 ^Ab^	15.55 ± 0.34 ^Ab^
b*-value				
Day 0	39.22 ± 0.66 ^Ab^	39.22 ± 0.66 ^Ac^	39.22 ± 0.66 ^Ac^	39.22 ± 0.66 ^Ac^
Day 5	38.82 ± 1.17 ^Ab^	38.76 ± 0.70 ^Ac^	38.99 ± 0.75 ^Ac^	38.92 ± 0.53 ^Ac^
Day 10	40.00 ± 0.51 ^Aab^	39.41 ± 0.58 ^Ac^	39.42 ± 0.43 ^Ac^	39.52 ± 0.83 ^Ac^
Day 15	41.74 ± 0.57 ^Aa^	40.29 ± 0.53 ^Bbc^	39.84 ± 0.41 ^Bbc^	40.20 ± 0.57 ^Bbc^
Day 20		41.64 ± 0.38 ^Aab^	41.20 ± 0.46 ^Aab^	41.55 ± 0.47 ^Aab^
Day 25		42.55 ± 0.49 ^Aa^	42.34 ± 0.93 ^Aa^	42.42 ± 0.36 ^Aa^

^A,B^ These values indicate significant differences between the various packaging treatments after the same storage time (*p* < 0.05). ^a–c^ These values indicate significant differences for the same packaging treatment after various storage times (*p* < 0.05). PAL: the palletized smoked chicken legs; M20, M60, and M100: the modified atmosphere packaged smoked chicken legs with CO_2_ concentration of 20%, 60%, and 100%, respectively.

**Table 2:** Number of total reads, observed operational taxonomic units (OTUs), diversity indices (Shannon and Simpson), and diversity richness (Chao1 and ACE) for the 16S rRNA amplicons of the control sample (fresh smoked chicken legs, Day 0) and the variously packaged samples at Day 25.

Storage Time	Treatment	Total Reads	Observed OTUs	Shannon	Simpson	Chao1	ACE	Good’s Coverage
Day 0	Control	7207	133	3.44	0.68	138.46	140.09	0.996
Day 25	M20	8744	74	1.89	0.45	86.83	96.99	0.994
	M60	9195	91	3.84	0.85	91.03	91.76	0.999
	M100	9584	75	3.70	0.86	82.09	84.64	0.996

M20, M60, and M100: the modified atmosphere packaged smoked chicken legs with CO_2_ concentration of 20%, 60%, and 100%, respectively.

## Data Availability

The data presented in this study are available within the article.
